# Evidence for the Efficacy and Safety of Tigecycline for the Treatment of Ventilator-Associated Pneumonia in Preterm Infants: A Systematic Review

**DOI:** 10.3390/life16091502

**Published:** 2026-09-08

**Authors:** Gorana Nedin Ranković, Dane Krtinić, Aleksandar Nikolić, Nada Pejčić, Nemanja Dimić, Nikola Milenković, Iva Binić, Branislava Ranković

**Affiliations:** 1 Department of Pharmacology with Toxicology, Faculty of Medicine, University of Niš, 18000 Niš, Serbia; 2 Clinic of Oncology, University Clinical Center Niš, 18000 Niš, Serbia; dane.krtinic@medfak.ni.ac.rs; 3 Department of Surgery and Anesthesiology with Reanimatology, Faculty of Medicine, University of Niš, 18000 Niš, Serbia; dracanikolic@gmail.com; 4 Clinic for Anesthesia and Intensive Care, University Clinical Center Niš, 18000 Niš, Serbia; nada.pejcic@gmail.com; 5 Clinic for Anesthesiology, Reanimatology and Intensive Care, University Clinical Hospital Centre “Dr Dragiša Mišović-Dedinje”, 11000 Belgrade, Serbia; nemanjadimic1989@gmail.com; 6 Department of Infectious Diseases and Epidemiology, Faculty of Medicine, University of Niš, 18000 Niš, Serbia; milenkovic@dr.com; 7 Clinic for Infectious Diseases, University Clinical Center Niš, 18000 Niš, Serbia; 8 Department of Psychiatry, Faculty of Medicine, University of Niš, 18000 Niš, Serbia; ivabinic@gmail.com; 9 Clinic for Psychiatry, University Clinical Center Niš, 18000 Niš, Serbia; 10 Institute of Pathology, Faculty of Medicine, University of Ljubljana, 1000 Ljubljana, Slovenia; bibarankovic@gmail.com

**Keywords:** tigecycline, ventilator-associated pneumonia, preterm infant, neonate, multidrug-resistant Gram-negative bacteria, glycylcycline, off-label, antimicrobial stewardship

## Abstract

Background: Ventilator-associated pneumonia (VAP) is a common and serious nosocomial infection in mechanically ventilated preterm infants and is increasingly caused by multidrug-resistant (MDR) and extensively drug-resistant (XDR) Gram-negative organisms. Tigecycline, a glycylcycline with broad activity against many such pathogens, is not approved below 18 years of age and carries a boxed warning for excess mortality that is most pronounced in hospital-acquired and ventilator-associated pneumonia. Its role, if any, in preterm infants with VAP is undefined. Objectives: To systematically identify and appraise all human evidence on the efficacy (clinical cure, microbiological eradication, survival) and safety (adverse events, mortality) of tigecycline used to treat VAP or nosocomial pneumonia during mechanical ventilation in preterm infants and neonates. Methods: A PRISMA 2020 structured search of PubMed/MEDLINE, Cochrane CENTRAL, Scopus, trial registries, regulatory documents, Google Scholar and reference lists was designed without language or date restrictions. Eligible reports described tigecycline treatment of pneumonia/VAP in neonates or young infants; pediatric case series and syntheses were retained as contextual evidence. Because only case reports and small non-comparative series were anticipated, a narrative synthesis was pre-specified; JBI tools and GRADE were planned for appraisal and certainty. Results: No randomized controlled trial, controlled observational study, or study dedicated to tigecycline for VAP in preterm infants was identified. Direct evidence meeting the full eligibility criteria (preterm neonate, VAP specifically, separately extractable outcomes) was limited to two case reports of extremely preterm neonates with VAP successfully weaned after tigecycline-based salvage combination therapy. Six further neonatal/young-infant reports initially considered were, on full-text re-review, reclassified as contextual (not index) evidence because they described non-VAP infections (sepsis, or CNS infections such as ventriculitis/meningitis), non-preterm ages, or mixed-infection series without separable VAP data. Reported outcomes were generally favorable in published cases but are subject to severe selection and publication bias; thrombocytopenia, hypofibrinogenemia and hepatic enzyme elevation were the principal adverse signals, against a class-level mortality signal concentrated in VAP. Quantitative pooling was not appropriate. The overall certainty of evidence was very low. Conclusions: There is no direct, credible efficacy or safety evidence supporting tigecycline for VAP in preterm infants. Available data neither establish benefit nor exclude harm. Based on this very-low-certainty evidence and on regulatory/class-level safety data rather than on demonstrated efficacy, tigecycline warrants consideration only as a last-resort, combination salvage option for culture-confirmed pan- or extensively drug-resistant pathogens when no safer alternative exists, with intensive monitoring and, ideally, within a registry or trial. Adequately designed neonatal pharmacokinetic and comparative safety studies are urgently needed. Registration: PROSPERO CRD420261450972 (registered 14 July 2026).

## 1. Introduction

### 1.1. Rationale

Preterm infants requiring mechanical ventilation are among the most vulnerable patients in the neonatal intensive care unit (NICU). Ventilator-associated pneumonia (VAP) is one of the most frequent device-associated infections in this setting, reported at roughly ten episodes per 1000 ventilator-days, and is associated with prolonged ventilation, longer stay, chronic lung disease and increased healthcare costs [1]. Diagnosis is notoriously difficult in preterm infants because the clinical and radiographic features of VAP overlap with respiratory distress syndrome, bronchopulmonary dysplasia and other pulmonary pathologies, and no universally accepted neonatal VAP definition exists [1,2,3]. These uncertainties complicate both trials and everyday management.

A growing proportion of neonatal VAP is caused by Gram-negative organisms—notably *Klebsiella pneumoniae*, *Pseudomonas aeruginosa* and *Acinetobacter baumannii*—that are increasingly multidrug-resistant (MDR) or extensively drug-resistant (XDR). When carbapenems and polymyxins fail or cannot be used, clinicians face a near-empty formulary, and interest turns to agents such as tigecycline [4,5].

Tigecycline is a glycylcycline that evades common tetracycline-resistance mechanisms and has broad in vitro activity against many MDR Gram-positive and Gram-negative pathogens. It is licensed in adults for complicated skin and soft-tissue infection, complicated intra-abdominal infection and community-acquired bacterial pneumonia but not for hospital-acquired or ventilator-associated pneumonia [6]. Two considerations dominate any pediatric use. First, tigecycline is a tetracycline-class drug: during tooth development, it can cause permanent dental discoloration, and the class can reversibly inhibit bone growth—a decrease in fibula growth rate has been documented in premature infants exposed to tetracyclines—so use below eight years of age is generally avoided [6]. Second, a pooled analysis of adult trials showed an all-cause mortality imbalance with tigecycline that led to a boxed warning; the excess deaths were most apparent in patients treated for hospital-acquired and, in particular, ventilator-associated pneumonia [6,7]. Because pediatric efficacy and safety trials were never conducted, regulators advise against use below 18 years unless no alternative exists; the European Medicines Agency permits use from eight years only on specialist advice [4,6].

Against this backdrop, tigecycline is nonetheless used off-label as a drug of last resort in children and, occasionally, neonates with otherwise untreatable infections [4,8,9,10]. Prior pediatric syntheses have summarized heterogeneous experience across infection sites and ages [4,8], but none has focused on the specific and high-stakes question of VAP in preterm infants—the very indication in which the adult mortality signal is strongest and in which tetracycline-class skeletal and dental effects are most concerning. A focused systematic review is therefore warranted, if only to define the size and quality of the evidence gap and to guide responsible practice and future research.

### 1.2. Objectives

Using PRISMA 2020 methodology [11], we sought to answer the following question, framed as PICO:

**Population**—Preterm infants (and, given anticipated scarcity, neonates and young infants) receiving mechanical ventilation who developed VAP or nosocomial pneumonia;

**Intervention**—Systemic (or, where reported, local) tigecycline, alone or in combination;

**Comparator**—Any comparator or none;

**Outcomes**—Primary: clinical cure/improvement and all-cause mortality; secondary: microbiological eradication, duration of ventilation/stay, and adverse events (notably thrombocytopenia, hypofibrinogenemia, hepatotoxicity, pancreatitis, and dental/skeletal effects).

## 2. Methods

This review is reported in accordance with the PRISMA 2020 statement [11]. The completed PRISMA 2020 checklist appears in Appendix A.

### 2.1. Protocol and Registration (Item 24)

The protocol was registered on PROSPERO (CRD420261450972, registered 14 July 2026). No amendments have been recorded to date; any future amendments will be reported here and updated on PROSPERO. All planned database, registry, regulatory and hand searches described below have been completed (final search dates 16–17 July 2026); this manuscript reports the finalized review, not an interim protocol.

### 2.2. Eligibility Criteria (Item 5)

**Inclusion.** Primary (index) evidence: Any prospective or retrospective study, case series, or case report describing tigecycline administered specifically to a preterm neonate to treat VAP specifically during invasive mechanical ventilation, with at least one efficacy or safety outcome reported separately for the VAP episode. A report satisfying only part of this definition—e.g., a non-pneumonia infection (sepsis, meningitis, ventriculitis), a non-preterm age, or a mixed-infection series from which VAP-specific outcomes could not be separately extracted—does not qualify as index evidence and is classified below as contextual evidence only, regardless of its tigecycline content.

**Contextual evidence.** Pediatric case series and prior evidence syntheses that included neonates/infants, or that reported respiratory tract infections, were retained separately to inform safety, dosing and the certainty assessment but were not counted as index studies. This category also includes neonatal/infant reports of tigecycline use for non-VAP indications (sepsis, meningitis, ventriculitis), reports in infants outside the preterm/neonatal window, and mixed-infection series from which VAP-specific data could not be separated—all reclassified here from index evidence after full-text re-review against the criteria above; the two reports meeting full eligibility are summarized in Table 1, and reclassified/contextual reports in Table 2.

**Exclusion.** Studies confined to term neonates, infants and patients older than early childhood with no extractable neonatal/infant or respiratory data; animal, in vitro, or pharmacokinetic-only studies (used only for background); narrative reviews, editorials and conference abstracts without primary data; and duplicate reports of the same cohort.

### 2.3. Information Sources (Item 6)

The following sources were specified: PubMed/MEDLINE, Cochrane Central Register of Controlled Trials (CENTRAL), Scopus, Google Scholar; trial registries (ClinicalTrials.gov, WHO ICTRP, EU-CTR); regulatory sources (US FDA and EMA product information and safety communications); and hand searching (backward and forward citation) of reference lists of all included reports and relevant reviews. Embase and a dedicated Web of Science search were not performed and are noted as a limitation (Section 4.3); Scopus was used as the primary multidisciplinary index. No language or date restriction was applied; non-English reports were to be translated. The preliminary scoping search underpinning this manuscript was last run on 8 July 2026, and the complete search was run on 16 July 2026.

### 2.4. Search Strategy (Item 7)

A structured strategy combined controlled vocabulary and free-text terms for the intervention and population, for example (MEDLINE syntax; to be peer-reviewed using PRESS and adapted per database):

(tigecycline[MeSH] OR tigecycline[tiab] OR glycylcycline*[tiab]) AND (“pneumonia, ventilator-associated”[MeSH] OR ventilator-associated pneumonia[tiab] OR VAP[tiab] OR nosocomial pneumonia[tiab] OR respiratory tract infection*[tiab]) AND (infant, newborn[MeSH] OR neonat*[tiab] OR preterm[tiab] OR premature[tiab] OR infant*[tiab] OR NICU[tiab]).

**Figure 1:**
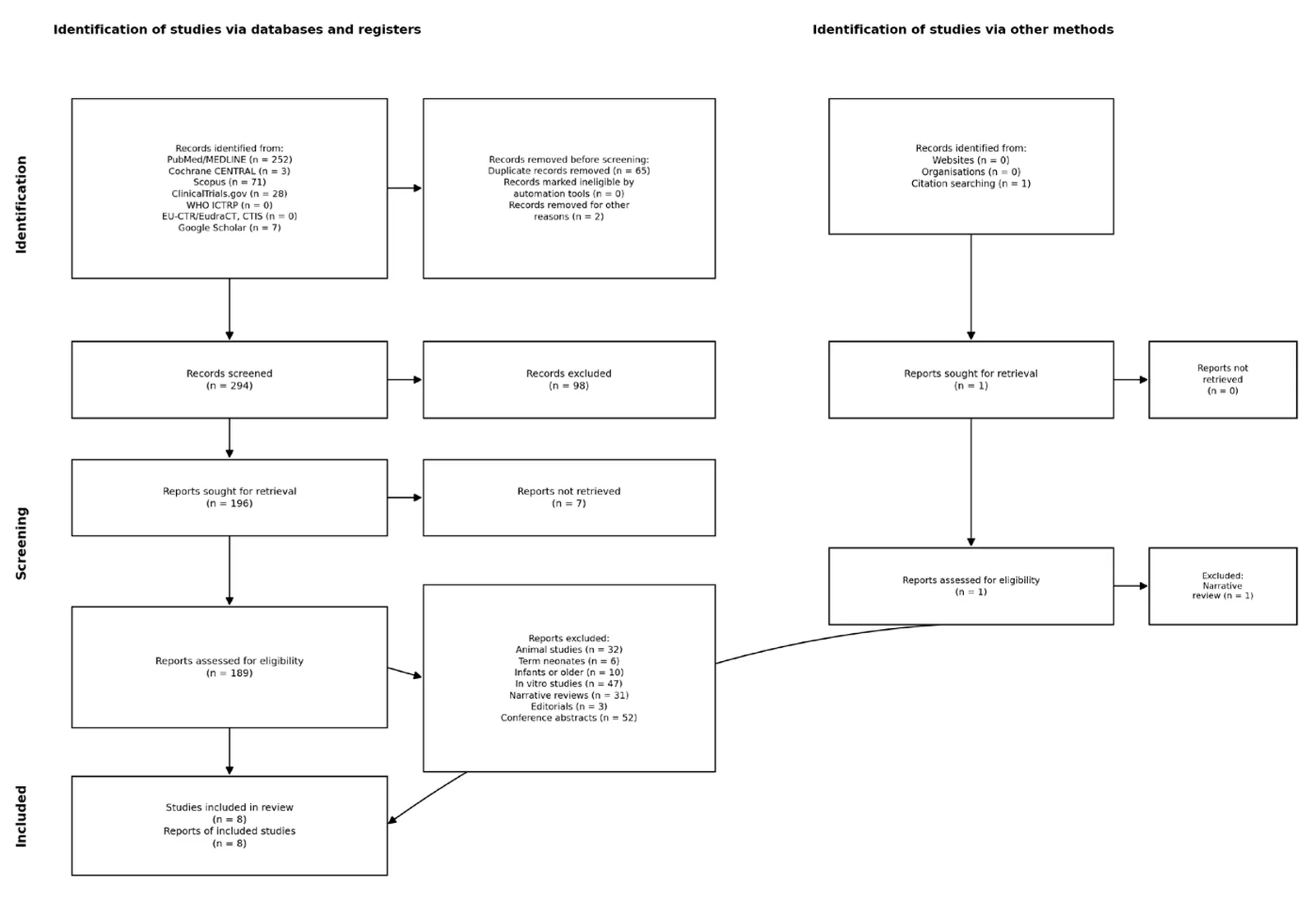
PRISMA 2020 flow diagram. The curved arrow indicates the direct path of eligible reports from full-text assessment into the included-studies box, bypassing the adjacent exclusion list.

A broader safety/efficacy strand omitting the pneumonia terms (tigecycline AND neonate/infant terms) was run to capture series in which pneumonia data are embedded. Full, ready-to-run strategies for every database—including both strands, controlled vocabulary, free-text terms and filters—are provided in Appendix B; the search dates and per-database hit counts are entered on execution and reconciled with Figure 1.

### 2.5. Selection Process (Item 8)

Two reviewers independently screened titles/abstracts, and then full texts, against the criteria in Section 2.2, with disagreements resolved by discussion between the two reviewers (no third reviewer was required). Records were managed and deduplicated in Microsoft Excel; title/abstract screening was performed manually, without a dedicated screening platform.

### 2.6. Data Collection Process and Data Items (Items 9, 10)

An extraction form captured: study identifiers and design; setting and dates; number and gestational age/birth weight/postnatal age of participants; infection site and whether VAP-defined; causative organism(s) and resistance profile; tigecycline dose, loading strategy, route, duration and co-antibiotics; and all efficacy and safety outcomes with timing. Two reviewers extracted independently; investigators were contacted for missing or unclear data. For continuous or count outcomes reported only graphically or descriptively, no imputation was performed.

### 2.7. Study Risk-of-Bias Assessment (Item 11)

Because the anticipated (and observed) evidence comprises case reports and non-comparative series, risk of bias/methodological quality was appraised with the Joanna Briggs Institute (JBI) critical-appraisal checklists for case reports and case series [12], applied independently by two reviewers. Randomized or comparative studies, if identified, were assessed with Cochrane RoB 2 or ROBINS-I as appropriate. The appraisal of the index reports is given in Table 3.

### 2.8. Effect Measures and Synthesis (Items 12, 13)

Efficacy measures of interest were proportions achieving clinical cure/improvement, microbiological eradication and survival; safety measures were proportions experiencing each adverse event. Because the eligible evidence is non-comparative, clinically and methodologically heterogeneous, and dominated by single cases, **a meta-analysis was neither valid nor planned**; results are synthesized narratively and tabulated by study, following the Synthesis without Meta-analysis (SWiM) reporting guideline [13] for grouping studies (index vs. contextual, Section 2.2), comparing outcomes qualitatively across reports, and handling heterogeneity by narrative description rather than statistical pooling. Had two or more sufficiently homogeneous comparative studies been found, a random-effects model with I^2^ heterogeneity assessment would have been considered [11].

### 2.9. Reporting-Bias and Certainty Assessment (Items 14, 15)

Reporting and publication bias are expected to be substantial and unquantifiable (favorable single cases are preferentially published); this is addressed qualitatively. Certainty in the body of evidence for each outcome was rated using the GRADE approach [14], starting low for non-comparative data and downgrading further for risk of bias, indirectness (population and outcome), imprecision and publication bias (Table 4).

## 3. Results

### 3.1. Study Selection (Item 16)

The study-selection process is summarized in the PRISMA 2020 flow diagram (Figure 1). Database and register searching identified 361 records (PubMed/MEDLINE 252, Scopus 71, ClinicalTrials.gov 28, Cochrane CENTRAL 3, Google Scholar 7, WHO ICTRP and EU-CTR/CTIS 0), and citation searching identified one further record. After removing 65 duplicates, automation-flagged records (0) and two records removed for other reasons, 294 records were screened; 98 were excluded, which left 196 reports sought for retrieval (7 not retrieved) and 189 assessed in full text. Of these, 181 were excluded (animal studies 32, term neonates 6, infants or older 10, in vitro studies 47, narrative reviews 31, editorials 3, conference abstracts 52), and a further single record identified via citation searching was excluded as a narrative review. **No randomized controlled trial, controlled cohort or case-control study, or any study dedicated specifically to tigecycline for VAP in preterm infants was identified.** Eight reports initially met broad neonatal/young-infant tigecycline criteria [9,10,15,16,17,18,19,20]; on full-text re-review against the eligibility criteria (Section 2.2), only two—Zeng et al. and Wang & Tan—described VAP specifically in preterm neonates with separately extractable outcomes and were retained as index evidence [18,19]. The remaining six neonatal/infant reports—describing non-VAP infections (sepsis, meningitis, ventriculitis) or infants outside the preterm window or lacking separable VAP-specific data [9,10,15,16,17,20]—together with nine pediatric series or syntheses were retained as contextual evidence (fifteen reports in total; Table 2) [4,5,21,22,23,24,25,26,27]. The final search and screening counts are reported in Figure 1.

Records excluded at full text (enumerated above by category) comprised reports without neonatal/infant or pneumonia-specific data, pharmacokinetic-only or in vitro studies, non-primary articles, and overlapping cohorts. Notably, several frequently cited pediatric tigecycline studies [5,8,21,22] were not eligible as index evidence because they either did not include neonates/preterm infants or did not report pneumonia outcomes separately; they are discussed as contextual evidence only.

### 3.2. Characteristics of Included Studies (Item 17)

Table 1 summarizes the two index reports meeting the full eligibility criteria (preterm, VAP-specific, extractable outcomes); Table 2 summarizes the fifteen contextual reports (six reclassified neonatal/infant reports plus nine pediatric series or syntheses). In both index cases, tigecycline was used as salvage combination therapy for culture-confirmed XDR Gram-negative VAP after failure of carbapenem- or polymyxin-based regimens, at doses of 1.2–2.5 mg/kg/day—lower than extrapolated older-child regimens, which reflects the absence of validated neonatal pharmacokinetics. Across the broader contextual evidence (Table 2), tigecycline was likewise used almost exclusively as salvage combination therapy for MDR/XDR Gram-negative infection, at similar empirical doses.

**Table 1:** Index evidence—Tigecycline for pneumonia/VAP or MDR infection in neonates and young infants.

Study (Country)	Design; N	Population/Infection	Pathogen; Tigecycline Use	Efficacy Outcomes	Safety Outcomes
Zeng et al., 2025 (Taiwan) [18]	Case report; 1 (of 2 cases)	26 + 4-week, 900 g extremely preterm neonate; VAP due to XDR A. baumannii	XDR A. baumannii (tigecycline MIC 2 µg/mL); IV tigecycline 2.5 mg/kg/day replaced meropenem after failure of meropenem + aerosolized colistin	Gradual respiratory improvement; successful ventilator weaning and extubation; discharged without oxygen dependence	No adverse effects reported for this case (10-day course)
Wang & Tan 2026 (China) [19]	Case report; 1	25 + 5-week, 800 g extremely preterm neonate (twin); on invasive MV from birth; CRE *E. coli* VAP first cultured on ventilator day 11	CRE *E. coli* (meropenem-resistant, aztreonam-sensitive); after amikacin + aztreonam caused nephrotoxicity, salvage combination started day 24: aztreonam 30 mg/kg q12 h + tigecycline 1.2 mg/kg q12 h (initial double dose) + nebulized colistin + fluconazole	After 17 days combination therapy: WBC/CRP/creatinine normalized, sputum cultures negative, gradual respiratory improvement on ventilator support	No adverse drug events during hospitalization; clinician-planned monitoring for tooth discoloration and renal effects

VAP = ventilator-associated pneumonia; XDR/MDR = extensively/multidrug-resistant; IV = intravenous. All values are as reported in the primary sources and require independent verification.

**Table 2:** Contextual pediatric evidence (not index studies).

Study (Country)	Design; N	Population	Key Efficacy Findings	Relevance/Caveats
Sharland et al., 2019 [4]	Evidence synthesis	Children with MDR infections (mixed ages/sites)	Summarized generally favorable but low-quality pediatric experience	Prior synthesis; not neonatal/VAP-specific
Kontou et al., 2023 [24]	Evidence synthesis	Neonates specifically (not mixed ages); MDR-GN infections	Narrative review of newer (CAZ-AVI, MER-VAB, IMI-REL) and repurposed (colistin, tigecycline, fosfomycin) agents; notes scarce neonatal trial data, off-label use	More recent, neonate-focused synthesis; not VAP-specific, no new primary cases
Chen et al., 2020 (China) [25]	Retrospective; 13 (of 1177 screened)	Pediatric liver transplant recipients; CRGNB infection (intra-abdominal, VAP, bloodstream)	TGC 2 mg/kg q12 h, mean 10.1 ± 5.1 days; clinical efficacy 84.6% (11/13); pathogen eradication 69.2% (9/13); no serious adverse event; K. pneumoniae 69%, A. baumannii 31%	Transplant recipients, not neonates; VAP one of three infection sites, not isolated
Lin et al., 2020 (China) [26]	Retrospective; 24	Critically ill children with VAP; median age 8 months (range 27 days–6.75 years)	TGC as salvage combination therapy; loading 1.5 mg/kg then 1 mg/kg q12 h in 70.8%; median duration 10.75 days; positive clinical response reported	Directly VAP + tigecycline-focused but mixed pediatric ages; in-hospital mortality 41.7% (10/24), mostly congenital heart disease; not neonatal-specific
Ozkaya-Parlakay et al., 2020 (Türkiye) [27]	Retrospective; 36 (7 neonates)	Children aged 14 days–18 y with MDR bacteremia (7 in newborn period, 19 burn patients)	TGC combination therapy in all cases, median 13 days; microbiological eradication 75%, clinical response 83%; all organisms carbapenem-resistant	Mortality 36% (13/36), 7 deaths from tigecycline-resistant breakthrough infection; neonatal subgroup not separately reported; not VAP-specific
Song et al., 2018 [21]	Retrospective; critically ill children	Post-surgical MDR/XDR infections	Salvage tigecycline associated with clinical response in a proportion of children	Surgical ICU; site-mixed
Ye et al., 2018 [22]	Retrospective; 37	Children with hematologic malignancy and infection	Improvement in ~49%	Immunocompromised; site-mixed
Avcu et al., 2025 (Türkiye) [5]	Retrospective; 67	Hospitalized children (median 6 y); LRTI commonest (~30%)	Clinical response ~66%; microbiological response ~63%; failure ~34%	Older children; LRTI includes non-VAP; no major adverse events reported
FAERS tetracyclines pharmacovigilance 2024 [23]	Disproportionality analysis	Pediatric adverse event reports for tetracyclines incl. tigecycline	Signals dominated by GI and general/administration-site disorders	Signal, not incidence; confounded reporting
Kanjanapattanakul & Nilwalaikul 2020 (Thailand) [9]	Retrospective case series; ~82 neonates	NICU neonates with XDR-organism infection; pneumonia was the commonest indication (~41%), septicemia ~33%; mixed infection sites, not VAP-isolable	Overall survival ~87%; no mortality difference between the two doses (*p* ≈ 0.44)	Mixed-infection series; VAP-specific outcomes not separately extractable—reclassified from index to contextual evidence
İpek, Günel & Özbek 2019 (Türkiye) [10]	Case series; 4 newborns	Newborn infants with XDR K. pneumoniae sepsis, not pneumonia/VAP	All four reported successfully treated	Sepsis, not VAP; reclassified from index to contextual evidence
Pratheep et al., 2019 (India) [15]	Case report; 1	27-week, 1028 g preterm neonate; XDR A. baumannii ventriculitis (CNS infection), not VAP	Clinical/microbiological response reported after intraventricular tigecycline	CNS infection, not pneumonia/VAP; reclassified from index to contextual evidence
Peng et al., 2018 (China) [16]	Case report; 1	3-month-old infant (not a preterm neonate); MDR K. pneumoniae pneumonia	Recovery reported	Age exceeds preterm/neonatal scope; reclassified from index to contextual evidence
Krasnanova & Kovacikova 2020 [17]	Case report; 1	5-month-old infant (not a preterm neonate); MDR P. aeruginosa sepsis with RSV bronchiolitis, not VAP	Inflammatory markers fell within 48 h; survived 13-day course	Age and infection type outside scope; reclassified from index to contextual evidence
Arora et al., 2024 (India) [20]	Case report; 1	34 + 3-week preterm neonate; MDR K. pneumoniae meningitis/ventriculitis (CNS infection), not VAP	Discharged day 42; no abnormal movements; follow-up for HIE2/meningitis sequelae	CNS infection, not pneumonia/VAP; reclassified from index to contextual evidence

LRTI = lower respiratory tract infection; GI = gastrointestinal; FAERS = US FDA Adverse Event Reporting System. Contextual studies inform safety and dosing but not the primary preterm VAP question.

### 3.3. Risk of Bias in Studies (Item 18)

Both index reports are single, non-comparative case reports and are at high risk of bias for questions of efficacy and safety (Table 3), itemized by JBI domain. Case reports cannot support incidence or effect estimates, regardless of how completely they are documented; they are retrospective, single-center, without control groups or adjustment, and vulnerable to selection, indication, survivorship and reporting biases. Favorable outcomes are systematically over-represented because unsuccessful single cases are less likely to be published. The contextual neonatal/pediatric series (Table 2) share the same structural limitations and additionally cannot contribute VAP-specific effect estimates, because none met the eligibility criteria for index evidence.

**Table 3:** Methodological appraisal of the two index reports (JBI critical appraisal checklist for case reports, itemized by domain).

Report	D1	D2	D3	D4	D5	D6	D7	D8	Overall Judgement
Zeng et al., 2025 [18]	Y	Y	Y	Y	Y	Y	Partial	Y	High risk of bias for causal inference (single case; no comparator)
Wang & Tan 2026 [19]	Y	Y	Y	Y	Y	Y	Y	Y	High risk of bias for causal inference (single case; no comparator)

D1 = patient demographics clearly described; D2 = patient history clearly described/timeline; D3 = clinical condition on presentation clearly described; D4 = diagnostic tests/methods and results clearly described; D5 = intervention(s)/treatment clearly described; D6 = post-intervention clinical condition clearly described; D7 = adverse events identified and described; D8 = takeaway lessons provided. Y = yes; Partial = partially reported. Individual domains reflect reporting completeness; the overall judgment of high risk of bias for causal inference reflects the single-case, non-comparative design itself, which no amount of reporting completeness can overcome.

### 3.4. Results of Individual Studies and Synthesis (Items 19, 20)

**Efficacy.** In the two index cases, tigecycline-containing salvage combination therapy was followed by gradual respiratory improvement, successful ventilator weaning and survival to discharge in both [18,19]. With only two uncontrolled cases, these outcomes cannot be interpreted as efficacy evidence: there is no comparator and no denominator beyond the cases themselves, and survivorship/publication bias cannot be excluded—a case ending in death is intrinsically less likely to be written up. The broader contextual evidence (Table 2) is consistent with the same pattern of favorable but uncontrolled outcomes in non-VAP or non-preterm tigecycline salvage use—for example, an overall survival of roughly 87% in a mixed-indication neonatal series [9] and successful treatment of four neonates with XDR K. pneumoniae sepsis [10]—but none of this contextual evidence can be attributed to VAP specifically, and none isolates an effect of tigecycline on VAP outcomes in preterm infants.

**Safety.** The most consistently reported adverse signals were hematological—thrombocytopenia and hypofibrinogenemia—together with reversible hepatic enzyme elevation and gastrointestinal intolerance; acute kidney injury and death in the neonatal series occurred in infants with pre-existing septic shock rather than being clearly attributable to the drug [6,9,10,23]. Crucially, the class-level, adult-derived mortality signal is concentrated precisely in ventilator-associated pneumonia [6,7], and tetracycline-class effects on developing teeth and bone are of specific concern in preterm infants [6]. No study was designed or powered to detect these harms in neonates. Because the data are non-comparative and heterogeneous, no pooled estimate was calculated (Section 2.8).

### 3.5. Reporting Biases and Certainty of Evidence (Items 21, 22)

Publication and reporting bias are judged as very serious: the literature is composed almost entirely of favorable single cases and small series, with no registered or negative studies to counterbalance them. When GRADE is applied, certainty in every outcome (clinical cure, microbiological eradication, survival, and each harm) is **very low**, which reflects the non-comparative design, serious indirectness for the outcomes drawing on contextual/regulatory/class evidence rather than the two direct index cases (Table 4), serious imprecision, and serious publication bias (Table 4).

**Table 4:** GRADE certainty summary, with direct (index) evidence distinguished from indirect (contextual/regulatory/class) evidence.

Evidence Type	Outcome	Evidence Base	Certainty	Main Reasons for Downgrading	Plain-Language Finding
Direct (2 index preterm-VAP cases)	Clinical cure/improvement	Zeng et al.; Wang & Tan [18,19] (case reports)	Very low	Non-comparative single cases; indirectness; imprecision; publication bias	Uncertain; cannot confirm benefit
Direct (2 index preterm-VAP cases)	Microbiological eradication	Zeng et al.; Wang & Tan [18,19] (case reports)	Very low	As above	Uncertain
Direct (2 index preterm-VAP cases)	All-cause mortality (preterm VAP)	Zeng et al.; Wang & Tan [18,19] (both survived)	Very low	Only 2 cases, both successful; severe publication bias toward favorable single cases	Cannot exclude harm; survivorship reporting bias likely
Indirect (adult VAP data)	All-cause mortality (class-level signal)	Adult tigecycline RCT pooled mortality imbalance; boxed warning	Very low (indirect)	Serious indirectness (adult population, not neonatal)	Possible harm cannot be excluded; signal concentrated in VAP
Indirect (contextual pediatric series + class data)	Thrombocytopenia/hypofibrinogenemia	Contextual pediatric/neonatal series (Table 2) + class pharmacovigilance data	Very low	Reporting bias; no denominators; indirectness	Plausible, monitoring warranted
Indirect (tetracycline-class data)	Dental/skeletal effects	Tetracycline-class developmental toxicity data (no preterm-VAP-specific follow-up)	Very low (indirect)	No neonatal follow-up data; extrapolated from class effects	Biologically expected; long-term risk

## 4. Discussion

### 4.1. Principal Findings and Interpretation (Item 23a)

This systematic review set out to determine whether tigecycline is effective and safe for VAP in preterm infants and found that **the direct evidence base is almost empty, limited to two extremely preterm VAP cases.** No controlled study, and no study dedicated to this population and indication, exists. What can be assembled is a small collection of retrospective neonatal series and individual case reports in which tigecycline was used as a last-resort, combination salvage agent for MDR/XDR Gram-negative infection—sometimes pneumonia—with generally favorable but wholly uncontrolled outcomes [9,10,15,16,17,18,19,20]. These reports establish only that tigecycline can be administered to neonates and that survival is possible; they cannot establish that it improves outcomes, and they are structurally incapable of detecting the harms that matter most here.

A separate, larger-scale source offers indirect support for the rationale behind these salvage attempts without closing the clinical evidence gap: a single-center retrospective cohort of 61 neonates meeting the VAP criteria (48 with complete data) found that susceptibility to amikacin, colistin, and tigecycline remained relatively preserved among the Gram-negative organisms (predominantly *Klebsiella pneumoniae* and *Escherichia coli*) causing neonatal VAP, even as susceptibility to first-line agents declined over the study period [28]. This is microbiological susceptibility data, not clinical outcome data—no patient in that cohort was necessarily treated with tigecycline—and it therefore explains why tigecycline is considered rather than providing evidence that using it improves outcomes.

Interpretation must be anchored by two external facts. First, in adults, the agent carries a boxed warning for excess mortality that is most pronounced in hospital-acquired and ventilator-associated pneumonia—the exact indication under review—plausibly related to low serum concentrations at standard dosing [6,7]. The manufacturer states explicitly that pediatric trials were not conducted because of this adult mortality signal [6], which explains, rather than merely coincides with, the absence of pediatric-specific evidence identified by this review. This mortality signal is not confined to VAP alone: a meta-analysis restricted to multidrug-resistant Acinetobacter baumannii infections—the pathogen implicated in several of the index reports here—found that tigecycline is associated with significantly higher in-hospital mortality and lower microbiological eradication than comparator regimens [29]. Second, tigecycline is a tetracycline; dental discoloration and reversible bone-growth inhibition are class effects of specific concern in the developing preterm infant, in whom no long-term follow-up data exist [6]. Favorable short-term case reports do not offset these considerations.

Our findings are consistent with, but more cautious than, prior pediatric syntheses that concluded that tigecycline “may be an option” when alternatives are exhausted [4,5,8]. Those syntheses aggregated mixed ages and infection sites; by focusing on preterm VAP, we expose how little of that reassurance transfers to the highest-risk indication in the most vulnerable patients.

### 4.2. Limitations of the Evidence (Item 23b)

Several limitations, mentioned individually above, are consolidated here: (1) The two direct index cases and the fifteen contextual reports are all non-comparative, retrospective, small, single-center and geographically clustered, precluding any effect or incidence estimate. (2) Tigecycline was given almost universally as a combination therapy, so no report can isolate tigecycline’s own contribution from that of co-administered agents. (3) Publication bias toward favorable outcomes is severe and structural: cases ending in death are intrinsically less likely to be written up, and this cannot be corrected for retrospectively. (4) Neonatal pharmacokinetic and pharmacodynamic data for tigecycline are essentially absent, so all reported dosing is empirical rather than validated. (5) The diagnostic criteria for VAP are heterogeneous and largely non-validated across the primary studies: no universally accepted neonatal VAP definition exists (Section 1.1), and, where extractable, the two index reports applied standard clinical–radiological–microbiological criteria (new or progressive infiltrate plus clinical signs of infection plus a positive lower respiratory culture during mechanical ventilation) without citing a validated neonatal-specific instrument; the contextual series report VAP, pneumonia or nosocomial infection under varying and often unstated definitions. This heterogeneity means that the “pneumonia”/“VAP” label is not directly comparable across reports and raises a genuine risk of misclassification bias, which should be borne in mind when interpreting Table 1 and Table 2. (6) Long-term dental, skeletal and neurodevelopmental outcomes are unreported in every included source.

### 4.3. Limitations of the Review Process (Item 23c)

The protocol has been registered with PROSPERO (CRD420261450972), but grey literature and non-English sources were not fully captured, and Embase and a dedicated Web of Science search were not performed (Section 2.3), which may have missed additional records. In addition, several potentially relevant studies were published only as conference abstracts or otherwise lacked extractable data and could not be included. Relevant reports could have been missed.

### 4.4. Implications for Practice, Policy and Research (Item 23d)

As a matter of systematic review conclusion, this evidence base does not establish tigecycline as an effective or safe option for VAP in preterm infants, and it does not permit an evidence-based recommendation either for or against its use. Separately, as clinical prudence derived from the adult regulatory warning and tetracycline-class safety data rather than from the (absent) preterm VAP efficacy evidence, clinicians who nonetheless consider tigecycline would reasonably restrict it to a last-resort, combination salvage role for culture-confirmed pan- or extensively drug-resistant pathogens for which no safer effective agent exists, after multidisciplinary infectious diseases and neonatology input and informed parental discussion, with close monitoring of platelet count, fibrinogen and liver enzymes; time-limited treatment; and reporting to pharmacovigilance systems. This is offered as clinical prudence, not as a conclusion the review evidence itself can support.

This recommendation should also be read alongside newer anti-Gram-negative agents increasingly used for MDR/XDR infections, none of which carries tigecycline’s VAP-specific mortality signal. Ceftazidime–avibactam, ceftolozane–tazobactam, meropenem–vaborbactam and imipenem–relebactam have accumulating neonatal case report and case series evidence with a favorable reported safety profile, per a dedicated systematic review of novel antimicrobials in newborns [30]; ceftazidime–avibactam in particular is the most-studied agent in this population. Cefiderocol has likewise been used successfully off-label in the newborn period for nosocomial/pneumogenic infection, including a recently reported case of neonatal nosocomial pneumonia [31], though data below 3 months of age remain confined to isolated case reports. Aztreonam–avibactam and fosfomycin have sparser neonatal data still, generally limited to case reports or combination use. Where in vitro susceptibility allows, these beta-lactam/beta-lactamase-inhibitor agents and cefiderocol may represent preferable first-line salvage options ahead of tigecycline precisely because they lack its adult mortality signal in VAP; a formal comparative recommendation, however, is beyond the scope of this tigecycline-focused review and would require its own systematic synthesis.

The mismatch between rising neonatal MDR/XDR Gram-negative infection and the near-total absence of neonatal evidence for salvage agents argues for coordinated neonatal antimicrobial trial networks, registries of off-label last-resort use, and regulatory incentives for neonatal pharmacokinetic study.

Priorities for future research are: (1) neonatal population pharmacokinetic/safety studies to define whether any dose achieves adequate lung exposure without toxicity; (2) a prospective registry capturing consecutive (including unsuccessful) neonatal uses to counter publication bias; and (3) comparative effectiveness studies against alternative last-resort regimens (e.g., optimized colistin-, cefiderocol- or newer β-lactam/β-lactamase-inhibitor-based therapy) where feasible.

## 5. Conclusions

No credible direct evidence supports the efficacy or safety of tigecycline for ventilator-associated pneumonia in preterm infants; direct evidence is limited to two uncontrolled case reports that neither demonstrate benefit nor exclude harm, set against an adult mortality signal that is strongest in VAP and tetracycline-class effects of particular concern in preterm infants. As a matter of clinical prudence rather than of evidence this review can establish, tigecycline would reasonably be reserved as a monitored, time-limited, last-resort salvage option only, and its use in this population should drive—not substitute for—the neonatal pharmacokinetic, safety and comparative studies that are urgently required.

## Data Availability

The extraction template, full search strategies (Appendix B), and screening decisions are available from the corresponding author upon reasonable request.

## Appendix A

**Table A1:** PRISMA 2020 checklist (item → location).

Item #	PRISMA 2020 Checklist Item	Reported in
1	Title—Identify as a systematic review	Title page
2	Abstract (PRISMA for Abstracts)	Abstract
3	Rationale	Section 1.1
4	Objectives (PICO)	Section 1.2
5	Eligibility criteria	Section 2.2
6	Information sources	Section 2.3
7	Search strategy	Section 2.4; Appendix B
8	Selection process	Section 2.5
9	Data collection process	Section 2.6
10a/10b	Data items (outcomes; other variables)	Section 2.6; Section 1.2
11	Study risk-of-bias assessment	Section 2.7; Table 3
12	Effect measures	Section 2.8
13a–f	Synthesis methods	Section 2.8 (narrative; meta-analysis not applicable)
14	Reporting-bias assessment	Section 2.9; Section 3.5
15	Certainty assessment (GRADE)	Section 2.9; Table 4
16a/16b	Study selection results; exclusions	Section 3.1; Figure 1
17	Study characteristics	Section 3.2; Table 1 and Table 2
18	Risk of bias in studies	Section 3.3; Table 3
19	Results of individual studies	Section 3.4; Table 1 and Table 2
20a–d	Results of syntheses	Section 3.4 (narrative; no pooling)
21	Reporting biases	Section 3.5
22	Certainty of evidence	Section 3.5; Table 4
23a–d	Discussion (interpretation; limitations; implications)	Section 4.1, Section 4.2, Section 4.3 and Section 4.4
24a–c	Registration and protocol	Section 2.1; Institutional Review Board Statement
25	Support	Funding
26	Competing interests	Conflicts of Interest
27	Availability of data, code, materials	Data Availability Statement

Checklist adapted from Page MJ et al., BMJ 2021;372:n71 [11] (CC BY 4.0). Alternating row shading is decorative only, to aid readability, and carries no additional meaning.

## Appendix B. Full Search Strategies

### Appendix B.1. General Notes

These strategies operationalize the two-strand approach described in Section 2.4. **Strand A (focused)** combines the intervention, condition (pneumonia/VAP/mechanical ventilation) and population (neonate/infant/preterm) blocks. **Strand B (sensitive)** combines only the intervention and population blocks, deliberately dropping the condition block so that neonatal/infant tigecycline case series in which pneumonia data are embedded—but not indexed under a pneumonia term—are still retrieved; every Strand B record is screened for extractable pneumonia/VAP data.

No language, publication-date, species or article-type limits are applied at the search stage; non-English records are translated and animal-only or clearly irrelevant records are removed during screening. The MeSH heading “Tigecycline” was introduced in 2010; the free-text terms (including “glycylcycline*” and the development code “GAR 936”) ensure the retrieval of earlier records. Each strategy was peer-reviewed with the PRESS 2015 checklist before execution, with the “Date last searched” and hit counts entered on the day of the run (Section Appendix B.6). No further re-runs are planned; these are the final search dates for this review.

### Appendix B.2. PubMed/MEDLINE

Box A1 Interface: NLM PubMed. Search builder/History.

Database: PubMed (MEDLINE). Date last searched: 16 July 2026. Limits/filters: none (no language, date, species or article-type limit). # = search line number; * = truncation (wildcard) symbol matching any word ending; this notation applies throughout Appendix B. #1 "Tigecycline"[Mesh] #2 (tigecycline[tiab] OR glycylcycline*[tiab] OR Tygacil[tiab] OR "GAR 936"[tiab]) #3 #1 OR #2 #4 "Pneumonia, Ventilator-Associated"[Mesh] #5 "Pneumonia"[Mesh] OR "Respiratory Tract Infections"[Mesh] OR "Healthcare-Associated Pneumonia"[Mesh] OR "Cross Infection"[Mesh] #6 ("ventilator-associated pneumonia"[tiab] OR VAP[tiab] OR "nosocomial pneumonia"[tiab] OR "hospital-acquired pneumonia"[tiab] OR HAP[tiab] OR pneumonia[tiab] OR "respiratory tract infection*"[tiab]) #7 "Respiration, Artificial"[Mesh] OR "Ventilators, Mechanical"[Mesh] OR ("mechanical ventilation"[tiab] OR "mechanically ventilat*"[tiab] OR intubat*[tiab]) #8 #4 OR #5 OR #6 OR #7 #9 "Infant, Newborn"[Mesh] OR "Infant, Premature"[Mesh] OR "Infant, Low Birth Weight"[Mesh] OR "Intensive Care Units, Neonatal"[Mesh] OR "Infant"[Mesh] #10 (neonat*[tiab] OR newborn*[tiab] OR "new born*"[tiab] OR preterm[tiab] OR pre-term[tiab] OR premature*[tiab] OR "low birth weight"[tiab] OR LBW[tiab] OR VLBW[tiab] OR ELBW[tiab] OR NICU[tiab] OR infant*[tiab] OR neonate*[tiab] OR baby[tiab] OR babies[tiab]) #11 #9 OR #10 #12 #3 AND #8 AND #11 (Strand A - focused) -> [63] records #13 #3 AND #11 (Strand B - sensitive) -> [252] records #14 #12 OR #13 (exported for screening) -> [252] records

### Appendix B.3. Cochrane CENTRAL (Cochrane Library)

Box A2 Records limited to trials. Search line by line in the Advanced Search/search manager.

Database: Cochrane CENTRAL. Date last searched: 16 July 2026. #1 MeSH descriptor: [Tigecycline] explode all trees #2 (tigecycline OR glycylcycline* OR Tygacil):ti,ab,kw #3 #1 OR #2 #4 MeSH descriptor: [Pneumonia, Ventilator-Associated] explode all trees #5 MeSH descriptor: [Pneumonia] explode all trees #6 (ventilator-associated pneumonia OR VAP OR nosocomial pneumonia OR hospital-acquired pneumonia OR pneumonia OR "respiratory tract infection*"):ti,ab,kw #7 MeSH descriptor: [Respiration, Artificial] explode all trees #8 (mechanical ventilat* OR intubat*):ti,ab,kw #9 #4 OR #5 OR #6 OR #7 OR #8 #10 MeSH descriptor: [Infant, Newborn] explode all trees #11 MeSH descriptor: [Infant, Premature] explode all trees #12 (neonat* OR newborn* OR preterm OR premature* OR "low birth weight" OR NICU OR infant* OR neonate*):ti,ab,kw #13 #10 OR #11 OR #12 #14 #3 AND #9 AND #13 (Strand A) -> [1] records #15 #3 AND #13 (Strand B) -> [2] records #16 #14 OR #15 -> [2] records

### Appendix B.4. Scopus

Box A3 Enter each strand in the Advanced Search box; no document-type or language limit.

Database: Scopus. Date last searched: 16 July 2026. S1 TITLE-ABS-KEY(tigecycline OR glycylcycline* OR Tygacil OR "GAR 936") S2 TITLE-ABS-KEY("ventilator-associated pneumonia" OR VAP OR nosocomial pneumonia OR hospital-acquired pneumonia OR pneumonia OR "respiratory tract infection*") S3 TITLE-ABS-KEY("mechanical ventilat*" OR intubat*) S4 S2 OR S3 S5 TITLE-ABS-KEY(neonat* OR newborn* OR preterm OR premature* OR "low birth weight" OR NICU OR infant* OR neonate* OR baby OR babies) S6 S1 AND S4 AND S5 (Strand A) -> [9] records S7 S1 AND S5 (Strand B) -> [68] records S8 S6 OR S7 -> [71] records

### Appendix B.5. Trial Registries, Regulatory and Grey Literature Sources

Trial registries (Date last searched: 16 July 2026): ClinicalTrials.gov (Advanced search/API): tigecycline AND (neonate OR infant OR preterm OR pneumonia) -> [14] WHO ICTRP: tigecycline AND pneumonia -> [46 records/32 trials] ; tigecycline AND infant/newborn/neonate/preterm (searched individually) -> [0 for each] EU CTR/EudraCT and CTIS (Clinical Trials Information System): tigecycline -> CTIS (2022-present): [4 records, 0 relevant]; EU-CTR/EudraCT (pre-2022): [21 protocols, 0 paediatric (Art.45)] Regulatory/grey literature (Date last searched: 16 July 2026): - US FDA: Drugs@FDA NDA 021821 (Tygacil, approved 15 June 2005 ; 23 label revisions to date; current label SUPPL-56, 03/31/2025). Label confirms: indicated only for age 18+; explicitly NOT indicated for hospital-acquired pneumonia incl. VAP (Limitations of Use, 1.4); Boxed Warning all-cause mortality +0.6% (95% CI 0.1–1.2), greatest difference in VAP patients (5.1–5.2); paediatric-relevant warnings on tooth discoloration/enamel hypoplasia and bone growth inhibition through age 8 (5.7–5.8). Section 8.4 (Pediatric Use) states verbatim: "Because of the increased mortality observed in TYGACIL-treated adult patients in clinical trials, pediatric trials of TYGACIL to evaluate the safety and efficacy of TYGACIL were not conducted" -- the explicit regulatory reason for the paediatric evidence gap. - EMA: EPAR/product information -> 2 authorised centrally: Tygacil (originator, authorised 24 Apr 2006, revision 36, last updated 22 Oct 2025) and Tigecycline Accord (generic, authorised 17 Apr 2020, revision 7, last updated 26 Sep 2025). Both remain in active authorised status. - Google Scholar: reviewed to saturation (no new relevant records for several consecutive pages), "tigecycline neonate pneumonia"/"tigecycline VAP infant" -> [~60 titles screened across both queries; 7 new relevant records] - Conference proceedings (last 10 yrs): ESPID, PAS, IDWeek, ECCMID/ESCMID Global -> [Not systematically searched; archives require conference login/registration not available to the authors. Considered a minor limitation, as relevant findings typically progress to published articles captured by the PubMed/Scholar searches above.] - Backward citation: reference lists of all included + reviews -> 0 additional records - Forward citation: performed via targeted web search (not formal Scopus/WoS "cited by", pending institutional access) for the key index reports -> 1 additional relevant record identified (Ozkaya-Parlakay et al. 2020 [27])

### Appendix B.6. Search Yield

**Source**	**Strand A**	**Strand B**	**Date Searched**	**Notes**
PubMed/MEDLINE	63	252	16 July 2026	Exported total = Strand B (252); Strand A is a subset
Cochrane CENTRAL	2	3	16 July 2026	—
Scopus	0	71	17 July 2026	—
ClinicalTrials.gov	28	28	16 July 2026	—
WHO ICTRP	0	0	16 July 2026	—
EU-CTR/CTIS	0	0	16 July 2026	—
FDA/EMA (regulatory)	n/a	n/a	16 July 2026	FDA label confirms NOT indicated for HAP/VAP; boxed warning mortality signal greatest in VAP patients. EMA: 2 authorized medicines (Tygacil + generic), no pediatric indication.
Google Scholar	~30 screened	~30 screened	16 July 2026	Reviewed to saturation, not fixed 200; 7 new relevant refs added (Zeng, Wang & Tan, Arora, Kontou, Uze Okay, Chen, Lin) [18,19,20,24,25,26,28]
Citation/hand search	1	1	16 July 2026	-
